# A Tale of Three Systems: Towards a NeuroImmunoEndocrine model of obesity

**DOI:** 10.1146/annurev-cellbio-120319-114106

**Published:** 2021-10-06

**Authors:** Conan J O O’Brien, Emma R Haberman, Ana I Domingos

**Affiliations:** 1 Department of Physiology, Anatomy, and Genetics, University of Oxford, Sherrington Building, Sherrington Rd, Oxford OX1 3PT

**Keywords:** Leptin, sympathetic, neuroimmune, innervation, adipose, macrophage

## Abstract

The prevalence of obesity is on the rise. What was once considered a simple disease of energy imbalance is now recognised as a complex condition perpetuated by neuro- and immuno-pathologies. In this review, we summarise the current knowledge of the neuroimmunoendocrine mechanisms underlying obesity. We examine the pleiotropic effects of leptin action, in addition to its established role in modulation of appetite, and discuss the neural circuitry mediating leptin action, and how this is altered with obesity, both centrally (leptin resistance) and in adipose tissues (sympathetic neuropathy). Finally, we dissect the numerous causal and consequential roles of adipose tissue macrophages in obesity and highlight key recent studies demonstrating their direct role in organismal energy homeostasis.

## Introduction

Obesity is a growing pandemic. Approximately one quarter of the world’s adult population classified as overweight, and over half a billion adults worldwide are obese (*Obesity and overweight*). Obesity predisposes individuals to a plethora of life-threatening diseases, including coronary artery disease, atherosclerosis, hypertension, type 2 diabetes, cancer, and multiple autoimmune disorders (Chu et al. 2018; Versini et al. 2014). Obesity itself fulfils the criteria for consideration as a disease; it causes disorder in the structure and function of the organisms it affects and manifests by distinguishing symptoms. Fundamentally, it is a disease of energy imbalance. A long-term positive energy balance, in which energy intake reigns over expenditure, causes an excess of energy stored as triglyceride in adipose tissue (AT). However, this overly simplistic characterisation belies the complexity of the condition.

Recent decades have brought with them the revelation that adipose tissues provide input signals to the neural circuits that regulate energy storage and expenditure (Larabee et al. 2020). Additionally, fat tissues are a reservoir for stromovascular cells, nerve tissue, and immune cells. The immune cell component, principally macrophages, mediates many of the adverse effects of the obese condition, directly controlling adipose tissue mass (Camell et al. 2017; Pirzgalska et al. 2017; Weisberg et al. 2003, 2006; Wolf et al. 2017). For this review we will focus on the contribution of macrophages, as the chief immune component of adipose tissue, to the pathophysiology of obesity. Moreover, adipose tissues are bona fide endocrine and immunomodulatory organs which engage in paracrine and endocrine signalling through the production of adipokines, including adiponectin (Fantuzzi 2013), various cytokines (Fain et al. 2004), and perhaps most notably, leptin (Fain et al. 2004; Friedman 2019; Kershaw & Flier 2004; Larabee et al. 2020).

**Figure 1:**
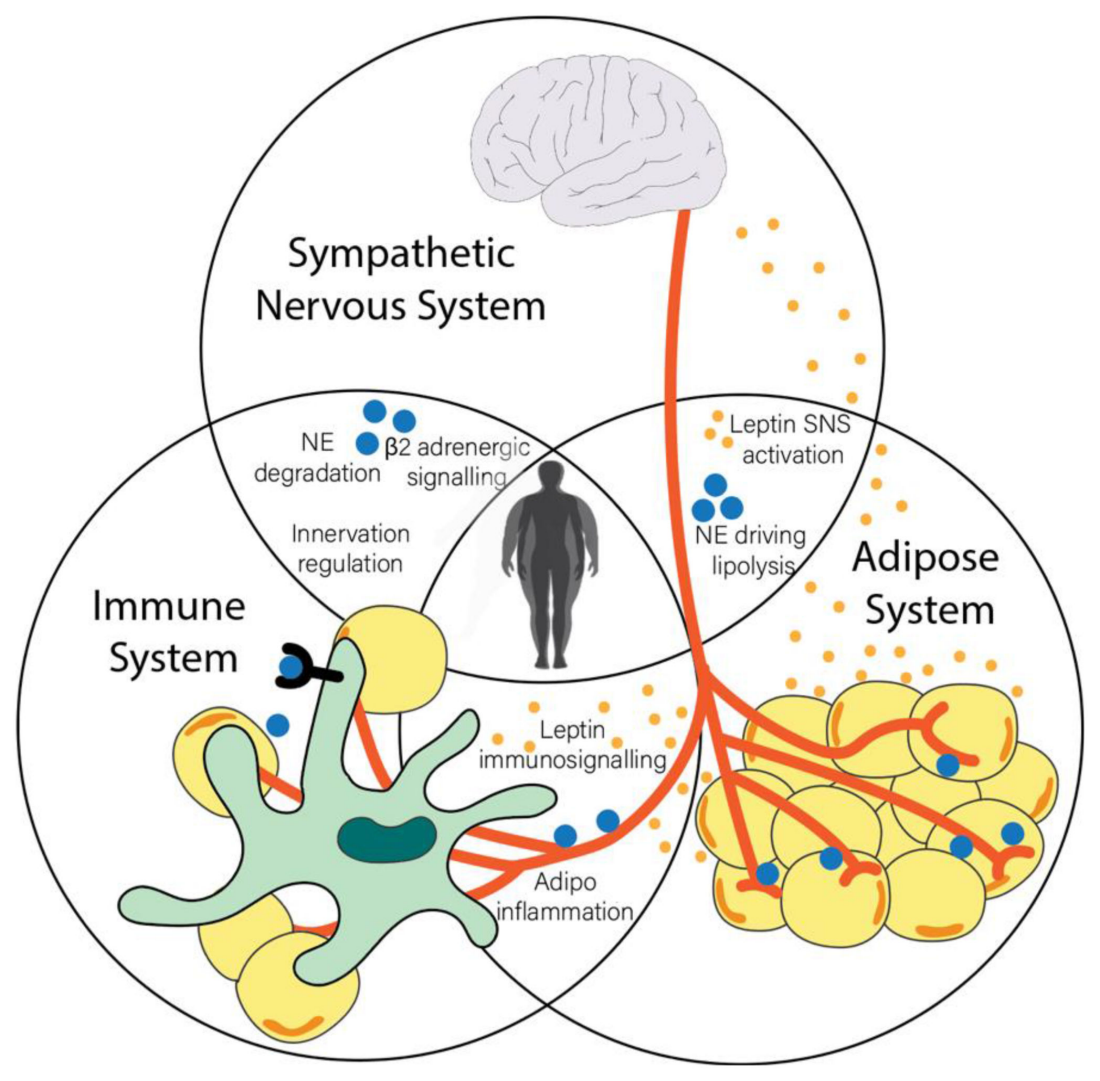
The systemic triad regulating energy expenditure. The sympathetic nervous, immune, and adipose systems converge in bi- and tri-directional manners to regulate systemic metabolism in humans. Adrenergic signalling (norepinephrine; NE), emanating from sympathetic nerve fibres, has various immunomodulatory effects and triggers lipolysis in adipose tissue. Sympathetic fibre-associated macrophages (SAMs) catabolise NE to inhibit this process and thereby inhibit weight loss. Adipose tissue-derived leptin is the afferent signal in a neuro-endocrine negative feedback loop that controls appetite and feeding. Leptin also has demonstrable energy expending effects in the periphery as evidenced by pair-feeding studies and is a potent immunomodulator of various innate immune cells. The adipose tissue itself is a dynamic endocrine organ, populated largely by macrophages in the obese state which promote inflammation and predispose to insulin resistance and type 2 diabetes.

This discovery of the *ob* gene and its product leptin in 1994 (Zhang et al. 1994) was the culmination of a decade’s long search for a blood-borne factor regulating nutrient intake and metabolism (Friedman 2019). The discovery changed our perception of adipose tissue from an energy storage depot to an endocrine organ and instigated a revolution in our understanding of obesity as a neuroendocrine condition (Figure 1). Accordingly, leptin levels are elevated in obesity in both mice and humans, and decline with weight loss (Ahrén 1999; Maffei et al. 1995). It is now widely acknowledged that leptin acts as the afferent signal in a negative feedback loop which regulates adipose tissue mass by reducing food intake (Campfield et al. 1995; Friedman 2019; Halaas et al. 1995; Maffei et al. 1995; Pelleymounter et al. 1995). However, it is becoming increasingly apparent that the effect of leptin on weight loss goes beyond behavioural modulation of appetite. The recent discovery of the sympathetic neuro-adipose junction closed the neuroendocrine negative feedback loop of leptin action in the brain. Sympathetic neurons constitute leptin’s efferent effector arm, which can directly mediate white adipose tissue lipolysis and thermogenesis (Zeng et al. 2015).

## Leptin: To infinity and beyond satiety

Leptin’s role in regulating energy balance was cemented by landmark studies demonstrating that leptin administration causes weight loss and reductions in adipose tissue (but not lean) mass in leptin-deficient *ob/ob* mice and diet-induced obese (DIO) mice, effects that are absent in leptin receptor-deficient *db/db* mice (Campfield et al. 1995; Halaas et al. 1995; Pelleymounter et al. 1995). Today, leptin’s commonly cited mechanism of mediating energy balance is through the central regulation of appetite and food intake (Friedman 2019). Indeed, the signalling isoform of the leptin receptor, ObRb, is most highly expressed in the arcuate nucleus of the hypothalamus (Baskin et al. 1999; Mercer et al. 1996), and central intracerebroventricular infusion of leptin into obese *ob/ob* and diet-induced obesity (DIO) mice causes a reduction in food intake and body weight in comparison to vehicle-treated controls; an effect that is absent in *db/db* mice (Campfield et al. 1995). Moreover, the neuronal deletion of ObRb in mice enhances adiposity, glucose intolerance, and food intake compared with ObRb^+^ controls (McMinn et al. 2005). However, among other symptoms human leptin deficiency manifests in low sympathetic tone, as measured by orthostatic hypertension and cold pressor response tests (Ozata et al. 1999), indicating that leptin’s effects also extend peripherally to sympathetically innervated tissues. In experiments where food intake of vehicle-treated *ob/ob* mice is matched to that of leptin-treated *ob/ob* mice (pair-feeding), vehicle-treated mice lose only half the weight of their leptin-treated counterparts, despite equivalent food consumption (Levin et al. 1996; Rafael & Herling 2000). The fact that leptin treatment causes weight loss that is additive to caloric deficit indicates that leptin-mediated control of body weight depends on more than the central hypothalamic-mediated suppression of feeding and implies peripheral mechanisms of leptin-mediated energy expenditure. However, Prieur et al. found that pair-fed *ob/ob* mice lost the same amount of weight as mice treated centrally or peripherally with leptin. Additionally, they showed that leptin treatment, regardless of route of administration, induced changes to hepatic and peripheral lipid metabolism indistinguishable from pair-feeding (Prieur et al. 2008), suggesting that the effects are mainly attributable to leptin’s effect on food intake. Of note, the latest timepoint in this study was after nine days of leptin administration. Prior studies (Levin et al. 1996; Rafael & Herling 2000) showing differences in body weight and feeding between pair-fed and leptin-treated groups ran for between 13 and 75 days (Prieur et al. 2008). Thus, it appears that though the authors’ interpretations may differ, the findings are compatible and reflect that the peripheral effects of leptin on body weight become apparent with prolonged observation.

Mechanistically, the effects of leptin on energy expenditure were long thought to be directly attributable to brown adipose tissue (BAT) UCP-1-dependent thermogenesis (Commins et al. 2001), and ‘beiging’: the uncoupling of respiration in white adipocytes resulting in a phenotype similar to that of brown adipocytes. Neuronal activation of ObRb^+^ neurons in the dorsomedial nucleus (DMN)/dorsal hypothalamic area (DHA) using Designer Receptors Exclusively Activated by Designer Drugs (DREADDS) drives energy expenditure via BAT thermogenesis and locomotor activity, independently of food intake (Rezai-Zadeh et al. 2014). Later studies would reveal the peripheral mechanism of leptin on body weight also directly impact the adiposity of the WAT (Zeng et al. 2015).

### Hypothalamic regulation of energy homeostasis

**Figure 2:**
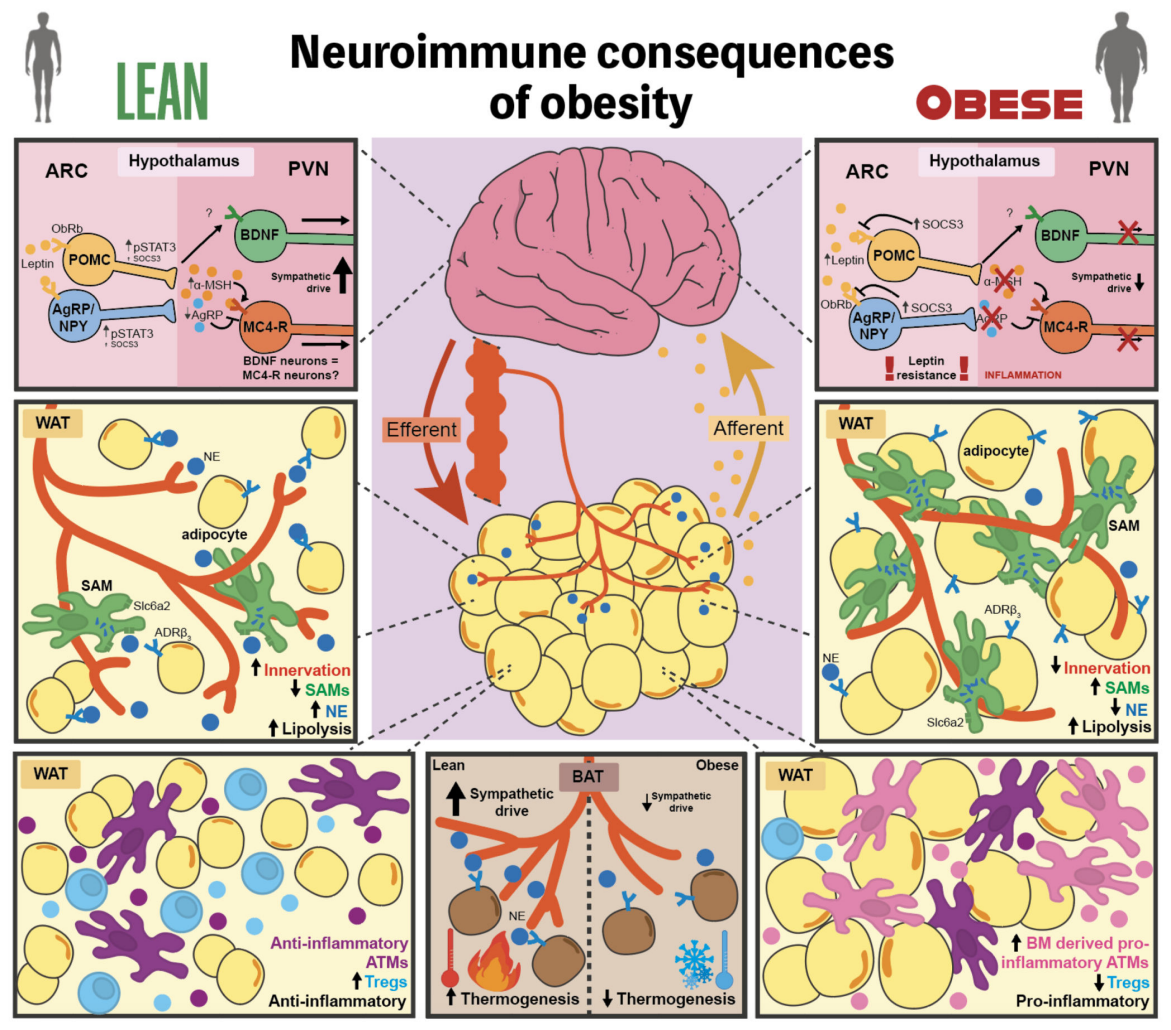
Neuroimmunity in Obesity. **Central panel:** The neuroendocrine loop of leptin action, where leptin constitutes the afferent arm and sympathetic neurons make up the efferent arm in the brain-adipose tissue axis. **Top panels, lean (left) and obese (right):** Leptin signalling results in the phosphorylation of STAT3 in POMC and AgRP neurons in the arcuate nucleus (ARC), which upregulate POMC and downregulate AgRP, respectively. Collectively, leptin signalling in the ARC results in activation of MC4-R^+^ neurons within the paraventricular nucleus (PVN), and a subsequent increase in sympathetic drive onto peripheral adipose tissues. BDNF neurons within the PVN also act downstream of ARC leptin signalling, and are required for the regulation of adipose tissue innervation. It is not clear whether BDNF and MC4-R neurons represent one or two populations within the PVN. Chronic leptin signalling in obesity increases SOCS3 – a known suppressor of leptin signalling – in POMC and AgRP neurons. Coupled with hypothalamic inflammation and ER stress, this is likely causative of central leptin resistance and impaired responses downstream of ARC leptin signalling. **Middle panels, lean (left) and obese (right):** Sympathetic neuron-Associated Macrophages (SAMs) act as a sink for norepinephrine (NE) in adipose tissues through Slc6a2-mediated uptake and MAOA degradation of NE, modulating lipolysis in WAT. Few SAMs associate with the dense sympathetic AT innervation in the lean state. Sympathetic innervation is reduced in obesity, and SAMs are massively recruited to WAT neurons, reducing NE-mediated adipocyte lipolysis. **Bottom WAT panels, lean (left) and obese (right):** In the lean state, yolk sac-derived, adipose tissue macrophages (ATMs) and Tregs maintain an anti-inflammatory state in AT. Bone marrow-derived pro-inflammatory ATMs are recruited with obesity, initiating chronic, low-level adipose tissue inflammation. **Bottom BAT panel (centre):** In addition to reduced sympathetic drive, innervation of brown adipose tissue is reduced with obesity, resulting in a reduction in NE levels and a concomitant decrease in thermogenesis (left vs. right).

The hypothalamus consists of distinct nuclei, which integrate and respond to centrally and peripherally derived autonomic and neuroendocrine feedback signals, and is responsible for whole organism energy homeostasis through the regulation of appetite and sympathetic drive to the periphery (Myers & Olson 2012). Whilst several hypothalamic-acting, blood-borne hormones – including the active thyroid hormone (T3), oestrogen and glucagon-like peptide-1 (GLP-1) – have been reported to regulate obesity (Larabee et al. 2020), here we will focus on the dysregulation of leptin signalling within the hypothalamus. The long, signalling, ObRb isoform is present throughout the brain – with particularly dense expression in the arcuate nucleus (ARC), where ARC ObRb^+^ neurons represent ~75% of ObRb^+^ hypothalamic neurons (Münzberg et al. 2004; Scott et al. 2009). Exogenous leptin treatment in lean mice activates ObRb^+^ pro-opiomelanocortin (POMC) anorexigenic and agouti-related protein/ neuropeptide Y (AgRP/NPY) orexigenic neurons within the ARC, as marked by the phosphorylation of signal transducer and activator of transcription 3 phosphorylation (pSTAT3) – a robust marker of leptin signalling (Figure 2) (Münzberg et al. 2003). As a result, expression of POMC – and therefore its cleavage product α-melanocyte-stimulating hormone (α-MSH) – is increased within these anorexigenic neurons, whereas orexigenic ‘AgRP/NPY’ neurons downregulate AgRP and NPY (Kitamura et al. 2006; Korner et al. 1999, 2001).

Notably, an intact Paraventricular Nucleus (PVN) within the hypothalamus is required for leptin-mediated induction of satiety and increase in adipose tissue sympathetic drive, firmly planting the PVN as a major mediator of leptin mediated action (Haynes et al. 1997; Kannan et al. 1989; Leibowitz et al. 1981; Scarpace & Matheny 1998). Both POMC and AgRP/NPY ARC neurons project onto melanocortin 4 receptor (MC4-R)^+^ pre-sympathetic neurons in the PVN, where they collectively drive activation of downstream MC4-R^+^ neurons through the combined increase in MC4-R agonist α-MSH and decrease in MC4-R antagonist AgRP (Cowley et al. 2001; Korner et al. 2001; Schwartz et al. 1996b, 1997; Stephens et al. 1995). Recently, Wang and colleagues also described a direct role for BDNF^+^ neurons within the PVN, which act downstream of ARC leptin signalling (Wang et al. 2020). A PVN-specific ablation of BDNF signalling was sufficient to blunt WAT lipolysis and reduce WAT and BAT sympathetic innervation following acute leptin administration, resulting in a reduction in leptin-induced weight loss and highlighting the importance of BDNF^+^ PVN neurons in sympathetic AT innervation (Wang et al. 2020). Of note, it is unclear whether MC4-R^+^ and BDNF^+^ neurons downstream of ARC leptin signalling represent one or two populations within the PVN (Figure 2).

Furthermore, following subcutaneous (scWAT) injection of fluorescently tagged pseudorabies virus retrograde tracing, Wang and colleagues also demonstrated the presence of ObRb^+^ PRV^+^ neurons within the DMN and medial preoptic area (MPO) of the hypothalamus (Wang et al. 2020). Of the areas reported to mediate leptin signalling to WAT and BAT, only ablation of ARC leptin signalling was sufficient to alter sympathetic drive onto scWAT and brown adipose tissue (BAT) (Wang et al. 2020). However, the MPO – which lacks a proper blood-brain barrier (BBB) and may therefore be more sensitive to fluctuations in serum leptin – has previously been linked with leptin-mediated regulation of reproduction, body weight and temperature (Yu et al. 2018). In addition to revealing downstream targets of ARC ObRb^+^ AgRP and POMC neurons, this study also highlights the importance of ARC central leptin action not only in regulating lipolysis (WAT) and thermogenesis (BAT), but also in modulating the sympathetic innervation of these tissues – discussed later (Wang et al. 2020). However, the inability of POMC-ObRb deficient models to recapitulate Ob/Ob or Db/Db levels of diet-induced obesity (DIO) indicates a role for leptin signalling in energy homeostasis outside of the hypothalamic ARC-PVN circuit (Figure 2) (Balthasar et al. 2004; Wall et al. 2008).

### Central leptin resistance

A common feature of diet-induced obesity (DIO) is leptin resistance – the attenuation of hypothalamic leptin signalling thought to be due to chronic, central ObRb overstimulation (Koch et al. 2014). Whilst short-term high-calorie intake can be countered through a centrally-mediated increase in energy expenditure, the chronic intake of highly calorific foods results in an inability to sufficiently increase energy expenditure to reinstate energy balance, culminating in weight gain. Of note, the term leptin resistance is difficult to quantify, and is used in a context-dependent manner (Myers et al. 2012). Here we use this term to describe the inability of POMC and AgRP neurons within the ARC to phosphorylate STAT3 in response to acute increases in leptin concentration, a phenomenon accompanying chronically elevated serum leptin, which is likely a contributing factor in DIO (Münzberg et al. 2004). Although exogenous leptin administration is sufficient to normalise both body weight and adiposity in leptin-deficient animals and humans (Farooqi et al. 1999; Pelleymounter et al. 1995), this does not represent a viable therapeutic strategy for most DIO cases, where individuals are hyperleptinaemic and unable to increase energy expenditure in response to acute leptin stimulus (Münzberg et al. 2004). Leptin-responsive cells are found both centrally and peripherally, however, DIO-induced dysregulation of leptin signalling specifically within the ARC is thought to be significant in the onset of leptin resistance (Münzberg et al. 2004). Whilst chronically high-fat diet (HFD)-fed mice have increased baseline pSTAT3 levels in comparison with normal diet (ND)-fed controls, these mice have both a significantly higher body weight and are unable to upregulate pSTAT3 in response to exogenous leptin, indicating the saturation of ARC leptin signalling with serum hyperleptinaemia (Münzberg et al. 2004). In particular, Knight and colleagues elegantly demonstrated that chronic serum hyperleptinaemia is required for the onset of central leptin resistance by comparing leptin response in HFD-fed hyperleptinaemic WT mice and normoleptinaemic Ob/Ob mice chronically infused with levels of leptin comparable to ND-fed mice (Knight et al. 2010). While both experimental groups gained comparable body weight with HFD, only normoleptinaemic mice increased ARC pSTAT3 levels in response to exogenous leptin, indicating that leptin resistance is dependent on hyperleptinaemia and not increased adiposity alone (Knight et al. 2010). Consistent with hyperleptinemia being causal to obesity, the group of Philipp Scherer demonstrated that normalisation of hyperleptinemia is sufficient to restore leptin sensitivity, drive weight loss, and restore body weight homeostasis (Zhao et al. 2019). This demonstration involved the use of neutralising antibodies against leptin and/or temporally controlled knockdown of leptin. These manipulations could be seemingly counterintuitive, as leptin reduction would be expected to increase appetite. However, at system level, the neuroendocrine loop of leptin action is far from being linear and may well be reset through interference with its afferent arm. Consistent with this notion is the demonstration that facilitation of the efferent sympathetic arm in the neuroendocrine loop of leptin action also drives weight loss, independently of changes in food intake (Kim & Diano 2020; Mahú et al. 2020; Morris 2020). In this later case, amplification of the sympathetic efferent arm by *sympathofacilitator* drugs is sufficient to reset the neuroendocrine loop, and restore body weight homeostasis independently of alterations in food intake and locomotion (Kim & Diano 2020; Mahú et al. 2020; Morris 2020). *Sympathofacilitator drugs* do not enter the brain, but have an anti-obesity effect by promoting weight loss via thermogenesis, coupled to heat dissipation by peripheral vasodilation. Because they do not enter the brain, they also do not have the typical cardiovascular nor behavioural side effects of centrally acting sympathomimetic drugs. The first in class in this category is pegylated amphetamine, whose effect does not rely on conventional targets of amphetamine, but rather on beta 2 adrenergic receptors - a well-known mediator of smooth muscle relaxation (Kim & Diano 2020; Mahú et al. 2020; Morris 2020). Taken together, interfering with either the afferent or the efferent arms in the neuroendocrine loop of leptin action the brain can drive weight loss and shortcut leptin resistance.

The induction of factors that inhibit leptin signalling in POMC and AgRP/NPY neurons has been suggested as one possible cause of ARC leptin resistance. Suppressor of cytokine signalling 3 (SOCS3) is one such inhibitor which is upregulated in a leptin signalling-dependent manner in ARC neurons of mice after only 4 weeks of HFD; whilst short-term HFD-induced SOCS3 upregulation is reversed in AgRP neurons 1 day following a switch from HFD to ND (Bjørbæk et al. 1999; Gamber et al. 2012; Münzberg et al. 2004; Olofsson et al. 2013). Alternatively, a lentiviral mediated knockdown of SOCS3 within the hypothalamus is protective against diet-induced obesity in rats (Liu et al. 2011). Despite the lack of tissue specificity in hypothalamic SOCS3 knockdown-mediated protection against diet-induced obesity, collectively these studies firmly implicate SOCS3 as a molecular mediator of ARC leptin resistance. Interestingly, SOCS3 expression is also increased in immune and stromal cells in response to inflammation in several human tissues, bringing about the possibility that hypothalamic inflammation may also be a factor in the upregulation of SOCS3 in ARC neurons in DIO (Figure 2) (White et al. 2011). Furthermore, a study by Ozcan and colleagues revealed an obesity-dependent increase in endoplasmic reticulum (ER) stress within hypothalamic neurons, and found that tunicamycin-induced ER stress in the brains of lean mice was sufficient to block hypothalamic STAT3 phosphorylation, following IP leptin administration (Ozcan et al. 2009). Excitingly, they found that this impaired leptin responsiveness could be reversed in obese mice by pre-treating orally with a chemical chaperone to enhance ER function; thus giving rise to a novel class of leptin-sensitising, anti-obesity drugs targeting ER stress (Liu et al. 2015; Ozcan et al. 2009). Notably, whether hypothalamic inflammation and associated ER stress and autophagy disorders, which have been extensively linked with diet-induced obesity, are causative or a consequence of leptin resistance is yet to be determined (Figure 2) (Souza et al. 2005; Thaler et al. 2013; Zhang & Kaufman 2008).

Another possible cause of central leptin resistance was thought to be blood-brain barrier (BBB) dysfunction, which has implications in the know mechanism of leptin sensitisers (Liu et al. 2015). Leptin is too large to diffuse freely through the BBB and must therefore be transported from the plasma. Whilst intraperitoneal (IP) leptin has little to no effect on pSTAT3 within the ARC following chronic HFD, intracerebroventricular (ICV) leptin administration induces hypothalamic STAT3 phosphorylation, albeit blunted in comparison with ND-fed, ICV leptin-treated mice; revealing saturation of leptin transport across the BBB (El-Haschimi et al. 2000; Maness et al. 2000). Further supporting this idea, Schwartz et al found that patients with a higher BMI reach a maximal cerebrospinal fluid (CSF) plasma concentration, despite notably higher plasma leptin concentrations (Schwartz et al. 1996a). In 2005, Oh hypothesised this may be due to impaired BBB transport mediated by unsaturated fatty acids with obesity, however a recent study by Harrison and colleagues using tissue clearing and light-sheet microscopy of fluorescently tagged leptin provides evidence that chronic HFD does not reduce leptin accumulation within the hypothalamus, compared with ND controls (Harrison et al. 2019; Oh-I et al. 2005). Although, it is clear from positron emission tomography (PET) imaging that only a fraction of labelled leptin delivered peripherally enters the brain, with the majority localising to the kidneys (Ceccarini et al. 2009). Taken together, cell-intrinsic leptin insensitivity, caused at least in part by ER stress, appears to be a major driver of central leptin resistance in obesity. Hypothalamic responsiveness to peripheral, exogenous leptin is further impaired by the saturation of leptin transport across the BBB accompanying hyperleptinaemia (El-Haschimi et al. 2000; Maness et al. 2000).

## Adipose tissue innervation

Although not recorded in most medical textbooks, indirect evidence for adipose tissue (AT) innervation in animals and humans has been accumulating since the 1960s. A number of early studies predicted a functional role for sympathetic innervation of adipose tissue, despite a lack in evidence to show sympathetic AT innervation. Whilst electrical stimulation of AT nerve bundles induced BAT thermogenesis and WAT lipolysis, the opposite was true following ganglionic blockade (BOGDONOFF et al. 1960; Correll 1963; Goodner et al. 1967). Furthermore, the addition of epinephrine to in vitro cultures of primary white adipose tissues is sufficient to increase serum free fatty acids (FFAs), further indicating a role for catecholamines in AT lipolysis induction (Galton & Bray 1967). However, initial efforts lacked sympathetic neuron specificity and were unable to define a direct role for sympathetic innervation in WAT and BAT homeostasis (Bowers et al. 2004; Fredholm & Rosell 1968; Klingenspor et al. 1994). A substantial amount of work, predominantly in hamsters and rats, has gone towards mapping the central representation of adipose tissues in the brain, through retrograde transsynaptic pseudo rabies virus (PRV) tracing of neurons – highlighting that fat pads are differentially represented in the brain and should thus be considered independent organs with distinctive predicted efferent pathways (Bamshad et al. 1998, 1999; Bowers et al. 2004; Huesing et al. 2020; Nguyen et al. 2014; Pereira et al. 2017; Youngstrom & Bartness 1995). Although widely used in neuroscience, one significant consideration with retrograde tracing studies is the specificity of PRV labelling. It has long been assumed that PRV transmission is exclusively transsynaptic, however little evidence to this effect exists, and in fact, non-synaptic exocytosis has been reported along the dendrites of pyramidal neurons within the hippocampus (Patterson et al. 2010; Svoboda 2019). Whilst it is generally accepted that BAT sympathetic innervation stems from the stellate ganglia (François et al. 2019; Morrison et al. 2000), controversy still surrounds the innervation of scWAT and visceral WAT (vWAT), although the level of experimental evidence is similar for both white adipose tissues where more detailed ganglia-specific analyses are limited by the difficulty of dissecting individual sympathetic ganglia. Conflicting reports have suggested that sympathetic innervation of scWAT stems from either thoracic (T13) – lumbar (L1-3) or the coeliac ganglia (Giordano et al. 2006; Jiang et al. 2017; Youngstrom & Bartness 1995), although these studies were performed in hamster and rats/mice respectively, which may explain the observed differences. The previously mentioned PRV studies were revisited by Huesing and colleagues, who utilised state-of-the-art tissue clearing and 3D light sheet imaging of whole torsos, allowing the direct visualisation of PRV transduction within intact paravertebral sympathetic chains *in situ* (Huesing et al. 2020). Although PRV are polysynaptic, these images suggest that scWAT innervation stems from T12-L1 in mice, with pre-ganglionic acetylcholinergic neurons arising from T7-T10 of the spinal cord (Huesing et al. 2020). The recent development of glycoprotein (G)-deleted rabies virus (RVdG), which requires co-injection of the missing viral glycoprotein gene order to produce complete viral particles, allows monosynaptic transmission from targeted neurons (Callaway & Luo 2015; Wickersham et al. 2007). Novel intersectional approaches such as this may allow for more finite, step-by-step tracing of efferent brain-AT sympathetic pathways.

With indirect evidence for the action of sympathetic neurons on white adipose tissue, in 2015 Zeng et al. definitively proved a direct role for sympathetic neuron-derived NE on inguinal scWAT lipolysis (Zeng et al. 2015). First, using clearing techniques which render adipose tissues transparent, they first detected nerve bundles throughout the entire 3D inguinal scWAT pad, which they found could be dissected. The whole axonal tree of sympathetic neurons in adipose tissue was visualised *in situ* three years later, by coupling antibody staining with DISCO clearing techniques (Cao et al. 2018; Chi et al. 2018). Intravital two-photon microscopy of murine adipose tissue revealed direct contact between sympathetic neurons and white adipocytes – coining the term ‘sympathetic neuro-adipose junctions’ (Zeng et al. 2015). Further reinforcing the functional role of AT sympathetic neurons, optogenetic stimulation of TH^CRE^Rosa26^Chr2^ expressing neurons resulted in an acute increase in WAT NE and lipolysis, which translated into a reduction in fat pad size over a four-week stimulation period (Figure 2) (Zeng et al. 2015). Moreover, leptin-mediated increases in AT NE and lipolysis were inhibited following either acetyl cholinergic ganglionic block, nerve crush injury or diphtheria toxin-mediated TH^+^ neuron ablation, highlighting the sufficiency and necessity of intact sympathetic AT innervation in sympathetic-mediated WAT lipolysis in response to leptin (Zeng et al. 2015).

Co-staining of nerve fibres dissected from adipose tissue with TH and a pan-neuronal marker revealed molecular heterogeneity, with scWAT fibres containing both sympathetic TH^+^ and non-sympathetic TH^-^ axons (Zeng et al. 2015). Notably, neuron presence does not necessarily equate to a tissue-specific functional role – several neurons found within adipose tissues are thought to be *en passant* and serve no functional role in AT homeostasis. This heterogeneous population of neurons within single nerve bundles also highlights the lack in the specificity of previously mentioned ‘sympathetic’ denervation or electrical stimulation/ extracellular recording experiments. Interestingly, whilst reports of parasympathetic innervation of WAT have been contradictory: a series of PRV retrograde tracing and functional studies all claim the inexistence of parasympathetic WAT innervation (Bowers et al. 2004; Giordano et al. 2006; Youngstrom & Bartness 1995). Regarding efferent innervation, conclusive evidence exists for the role of sympathetic innervation in adipose tissue, whereas parasympathetic innervation has never been reported.

The extensive PRV tracing studies performed by Bartness and colleagues also suggest sensory WAT and BAT innervation. Injection of herpes-simplex virus (HSV) 129 – an anterograde transsynaptic tracer – into scWAT results in the labelling of the dorsal root ganglia (DRG) 48 hours post-injection, which contains most sensory neurons within the spinal cord (Song et al. 2009). Additional evidence from Niijima indicates increased activity of afferent WAT neurons following injection of leptin; although specific, leptin-mediated activation of afferent neurons may be difficult to determine due to the extracellular recordings performed on WAT nerve fibres (Niijima 1998). Furthermore, sensory neurons have also been linked with the modulation of periovarian WAT beiging and particularly in BAT thermogenesis, where scWAT administration of a B3-adrenoreceptor agonist is sufficient to increase BAT temperature – an observation which is lost with surgical scWAT denervation (Garretson et al. 2016). Of note, WAT surgical denervation is not sensory neuron-specific, and likely results in damage to surrounding sympathetic neurons and vasculature. A capsaicin-induced, specific loss in unmyelinated (C) or thinly-myelinated (A-delta) fibres in the WAT of hamsters has also been linked with increased adiposity, further suggestive of afferent neuronal inputs in the existing leptin-regulated neuro-endocrine loop controlling AT homeostasis (Shi & Bartness 2005). Taken together, the presence of a functional sympathetic neuro-adipose junction regulating AT homeostasis is irrefutable, as is the presence of sensory neurons within WAT (Zeng et al. 2015). Although evidence exists for a potential sensory afferent input into the central modulation of AT homeostasis, the exact mechanism of function of these neurons – which also likely represent TH^-^ neurons within AT nerve fibres reported by Zeng and colleagues – is yet to be determined (Zeng et al. 2015).

### Plasticity of adipose tissue innervation

Adipose tissue sympathetic neurons undergo extensive morphological changes during metabolic challenge, including densification of the sympathetic axonal tree with cold shock, which is known to increase sympathetic drive onto AT (Brito et al. 2008; Cao et al. 2018; Jiang et al. 2017; Murano et al. 2009). Conversely, adipose tissue sympathetic innervation is significantly reduced in Ob/Ob mice compared with lean controls, and also seems to be reduced in human scWAT with increased BMI (Figure 2) (Blaszkiewicz et al. 2019). Whilst exogenous leptin treatment fully rescues AT sympathetic neuropathy in Ob/Ob mice and stimulates concomitant weight loss, cold shock, and sympathetic output onto ATs, it is unable to increase BAT innervation (Blaszkiewicz et al. 2019; Wang et al. 2020). Young mice with higher activity levels show an increase in total scWAT innervation and in particular sympathetic innervation, compared with sedentary, age-matched controls (Blaszkiewicz et al. 2019). The sympathetic drive-induced increase of BDNF in WAT stimulates an increase in neuronal – and specifically sympathetic neuron – density (Wang et al. 2020). This observed AT sympathetic neuropathy is analogous to the reversible loss in sympathetic innervation with chronic HFD in the liver (Liu et al. 2021). These reports suggest several potential targets that could be exploited therapeutically to restore the sympathetic efferent arm of the neuroendocrine loop of leptin action in DIO. Notably, the advancement of tissue clearing and 3D imaging techniques have been fundamental to these observations, which were not possible before due to the potential for inconsistencies in conventional 2D imaging of amorphous adipose tissues (Chi et al. 2018). Collectively, this suggests that sustained obesity culminates in sympathetic neuropathy in both mice and humans, although local players mediating such changes remain unknown.

Morphologically, even in lean mice adipose tissue, innervation in scWAT and vWAT are not equivalent. Whilst scWAT nerve fibres are thicker and dissectible, it is not possible to dissect nerve fibres from vWAT due to their more delicate nature (unpublished observations). This is likely reflective of microenvironmental differences, and consistent with the idea that individual WAT pads are distinct adipose tissue organs. Interestingly, levels of PRDM16 – primarily linked with brown adipocyte differentiation and identity – in lean mice are higher in scWAT than vWAT, and an adipocyte-specific ablation of PRDM16 results in the conversion of scWAT towards a vWAT morphological and molecular phenotype (Cohen et al. 2014; Seale et al. 2007). Moreover, a later study in lean mice suggested a role for adipocyte-specific PRDM16 in the regulation of adipose tissue sympathetic neurite density – making this an exciting candidate in the context of obesity-related sympathetic neuropathy, due to the spontaneous obesity accompanying an adipocyte-specific PRDM16 knockout (Cohen et al. 2014). It is also not yet clear how AT neuropathy in obesity might affect sensory innervation or function, which may also contribute to central leptin response in addition to the neuroendocrine negative feedback loop of leptin action. Another interesting point is the intrinsic sensitivity of adipose tissue neurons to metabolic stress and proinflammatory cytokines, both of which are enhanced during obesity and are likely also, in part, responsible for the observed peripheral adipose tissue neuropathy phenotype, as observed by Liu and colleagues in the metabolically challenged liver (Liu et al. 2021).

Owing to the contribution of sympathetic-associated macrophages (SAMs) to obesity, a local neuroimmune or neuro-adipose contribution in the dysregulation of adipose tissue innervation is highly likely, reflecting obesity-related changes in adipose tissue microenvironment. SAMs are massively recruited onto sympathetic neurons in both Ob/Ob and DIO mice (Pirzgalska et al. 2017); their close-involvement with sympathetic neurons in these two models of obesity may make them an excellent candidate for the regulation of innervation, amongst other proximal immune and stromal cells (Mahlakõiv et al. 2019; Spallanzani et al. 2019a). Substantiating the possibility for immune regulation of adipose tissue innervation, Wolf and colleagues described spontaneous obesity and impaired BAT innervation in a mouse model with a macrophage-specific deletion of Mecp2 (Wolf et al. 2017). Here they suggest a mechanism by which the upregulation of plexin-A4 on CX3CR1+ Mecp2 knockout macrophages repels neurons in a Sem6a-dependent manner, diminishing sympathetic innervation of BAT (Wolf et al. 2017; Yaron et al. 2005). Furthermore, the role of macrophage-derived TNFα in Sarm1-mediated sympathetic neuropathy in the livers of mice with DIO further implicates immune cells in the modulation of innervation (Liu et al. 2021). Besides, Wang and colleagues report impairment in the leptin-mediated enhancement of inguinal scWAT and BAT sympathetic innervation following the central disruption of BDNF neuron signalling (Wang et al. 2020). Moreover, the presence of BDNF within the brain but not adipose tissue highlights the possibility of additional centrally-mediated regulation of sympathetic adipose tissue innervation (Nakagomi et al. 2015). Adipose tissue-derived NGF signalling has also been suggested as a mediator of sympathetic innervation, however, a mechanism detailing obesity-related perturbations of NGF in neuropathy is yet to be determined (Cao et al. 2018).

## Leptin as an immunomodulator

The leptin receptor is a transmembrane receptor belonging to the class I cytokine receptor family (Tartaglia,’ et al. 1995). Accordingly, leptin signalling has demonstrable immunomodulatory effects. It is important to note that, although many studies utilise unvalidated leptin receptor antibodies to identify LepR-expressing cells, detection of transcripts by RT-PCR or *in situ* hybridisation remains the gold standard of detection due to a lack of knockout-validated antibodies against the receptor. RT-PCR of human peripheral blood mononuclear cells (PBMCs; lymphocytes and monocytes) reveals that ObR is functionally expressed and that its levels negatively correlate with BMI (Tsiotra et al. 2000),(Gabay et al. 2001).

In vitro leptin stimulation of PBMCs induces proliferation and secretion of TNFα, IL-6, and IFNγ; all potent inflammatory cytokines (Zarkesh-Esfahani et al. 2001). Stimulation of isolated human monocytes with leptin induces proliferation and activation, also stimulating release of TNFα and IL-6 release (Santos-Alvarez et al. 1999). The apparent proliferative and pro-inflammatory responses induced by leptin receptor signalling are consistent with its canonical signal transduction pathway via STAT3, a common protein activated by numerous cytokines and growth factors (Hillmer et al. 2016).

Notably, irradiated mice reconstituted with *db/db*, rather than WT, bone marrow have lower weight, adiposity, and lower expression of macrophage M1-like inflammatory markers in adipose tissue in response to 16 weeks of HFD (Dib et al. 2014). One recent paper (Chen et al. 2000) highlighted that male and female macrophages differ intrinsically in their response to leptin. Chen and colleagues showed that male mice fed HFD accumulate ten times more macrophages in their adipose tissue depots than females, and that male macrophages were more inflammatory than their female counterparts. Surprisingly, using primary murine bone-marrow-derived macrophages the group also showed that male BMDMs were intrinsically more migratory than female’s, and revealed leptin was itself a chemoattractant for the male’s but not for female’s BMDMs (Chen et al. 2000). These findings raise important questions about the function of macrophages in human obesity. Given the roles for macrophages in promoting adipo-inflammation, insulin resistance, and in regulation of adipose tissue mass, a greater understanding of sex-dependent differences in human macrophage accumulation and function is necessary to fully comprehend how they contribute to pathology in females.

Leptin also has chemotaxis-modulating effects on neutrophils (Montecucco et al. 2006), and chemotaxis and cytokine-modulating effects on eosinophils (Wong et al. 2007) and basophils (Suzukawa et al. 2011). In human basophils, leptin has a chemotactic potency similar to that of classical basophil chemokines and promotes survival, degranulation, and the production of Th2 cytokines (Suzukawa et al. 2011). In DCs, leptin promotes differentiation, survival, and induction of Th1 responses, while diminishing Treg, and Th17 responses (Mattioli et al. 2005; Moraes-Vieira et al. 2014). Indeed, leptin receptor signalling in T cells is necessary for Th17 differentiation (Reis et al. 2015). Taken collectively, these findings highlight the pleiotropic immunomodulatory roles of leptin and adipose tissue, by extension. Given the abundance of both in the obese state, the contribution of leptin to the obesity-associated immunopathology should not be underestimated and warrants further investigation.

## Immunometabolism

Immunometabolism describes the mechanism by which innate immune cells regulate the systemic metabolism of an organism (Larabee et al. 2020). For this review we will focus on the most abundant immune cell in obese adipose tissue, the macrophage. The literature now supports roles for these cells in the initiation and maintenance of the obese state and the adipo-inflammation associated with obesity which contributes to the comorbidities of insulin resistance and T2D.

### Macrophages

Macrophages dominate the immune landscape of adipose tissue, comprising ~5% of adipose tissue leukocytes in the lean state and up to 50% in obesity (Figure 2) (Weisberg et al. 2003). The accumulation of macrophages in white adipose tissue has been observed in mouse models of obesity and obese humans over the last 20 years. Early immunohistochemical (IHC) staining of human adipose tissue identified cells positive for the pan-macrophage marker CD68, in visceral and subcutaneous fat samples. They were found more commonly in visceral than in subcutaneous fat (Bornstein et al. 2000), highlighting that not all adipose tissues are created equal, and it is likely that adipose tissue-specific mechanisms exist that regulate tissue-specific inflammation.

IHC and *in situ* hybridisation reveal that the macrophage genes CD68 and F4/80 are upregulated in the WAT of DIO, *db/db*, and *ob/ob* mice (Xu et al. 2003). Weisberg et al. profiled RNA expression in visceral fat depots of lean and obese mice and showed that the macrophage-associated genes *(Csf1r, Cd68)* positively correlate with body weight. Immunohistochemical and qPCR analysis demonstrated the presence of macrophages in adipose tissue of obese mouse models, and that these cells were pro-inflammatory (M1-like) in nature, expressing the pro-inflammatory mediators *Tnfa, iNos, and Il6*. Bone marrow transplantation studies suggest that the vast majority of adipose tissue macrophages, over 85%, are bone marrow-derived and Csf1-dependent (Weisberg et al. 2003).

Indeed, adipocytes secrete the primary ligand for C-C motif chemokine receptor 2 (CCR2; expressed on circulating monocytes), the monocyte chemoattractant CCL2 (MCP-1), and its expression increases in obesity (Sartipy & Loskutoff 2003). In vivo studies in which CCR2 is genetically ablated or overexpressed show that these cells mediate obesity, obesity-induced hepatic steatosis, adipose tissue inflammation, insulin insensitivity, and the associated metabolic syndrome (Figure 2) (Ito et al. 2008; Kamei et al. 2006; Kanda et al. 2006; Kim et al. 2016; Obstfeld et al. 2010). It is important to note that CCR2-dependent macrophages are monocyte-derived cells that are recruited to adipose tissue over time in response to chemotactic signals including CCL2. The ability to specifically target these cells experimentally through CCR2 has facilitated our understanding of this macrophage subset. The evidence above supports the notion that they are distinct from truly adipose tissue-resident macrophages – cells likely derived from the yolk sac or fetal liver during development and which undergo local self-maintenance (Ginhoux & Guilliams 2016). Understanding these tissue resident macrophages in obesity has proven more difficult, primarily due to a lack of molecular tools to specifically manipulate this population. The rise in number macrophages in adipose tissue during obesity is not only due to the recruitment and differentiation of CCR2-dependent blood monocytes (Figure 2). Visceral ATMs proliferate locally in at least a partially CCL2-dependent manner. This proliferation contributes to macrophage accumulation in AT independently of blood monocyte recruitment (Amano et al. 2014).

### Phenotypic switch of macrophages in obesity

Macrophages, whether recruited or tissue-resident, adopt tissue-specific characteristics. They possess the receptors and machinery to detect and respond to cytokines, adipokines, insulin, free fatty acids, hormones, pathogen and danger-associated molecular patterns, and other mediators. Collectively, these mediators influence macrophage phenotype and function. Adipose tissue macrophages (ATMs) are no exception.

Though simplistic, the M1/M2 macrophage polarisation paradigm has been usefully employed to characterise the change in murine macrophage phenotype in response to obesity (Figure 2). Studies have shown that diet-induced obesity induces a switch in the profile of ATMs from M2-like “alternatively activated’ macrophages to the classically activated M1-like pro-inflammatory phenotype that contributes to insulin resistance (Lumeng et al. 2007, 2008). Notably, macrophages in Ccr2-deficient mice more closely resemble M2-like macrophages from lean mice (Lumeng et al. 2007). These M2-like macrophages are beneficial as PPARγ and PPARδ-deficient mice, which have impaired M2 polarisation, are more prone to diet-induced obesity, inflammation and insulin resistance (Kang et al. 2008; Odegaard et al. 2007, 2008). Indeed, PPARγ mutation in humans is associated with insulin resistance, T2D, and liposystrophy (Jeninga et al. 2009). Genetic deletion of Trib1 impairs differentiation of M2-like macrophages in various organs, including adipose tissues. This is accompanied by reductions in adipose tissue mass and evidence of enhanced lipolysis, glucose intolerance and insulin resistance (Satoh et al. 2013).

The M1/M2 paradigm becomes less useful in humans due to a lack of specific membrane markers to identify macrophage. Proteomic analysis of obese human ATMs reveals they do not express markers associated with the classically activated M1 phenotype. Instead, their defining cell markers - ABCA1, CD36, and PLIN2 - are proteins involved in lipid metabolism – more so than the prototypical human M2 markers. The expression of these metabolic markers is associated with pro-inflammatory gene expression, correlates with BMI, and is regulated by distinct signalling pathways involving the intracellular mediators p62 and PPARγ. Notably, considerable overlap in the metabolically activated markers exists between mice and humans (Kratz et al. 2014). These metabolically activated ATMs promote dead adipocyte clearing through Nox2-dependent lysosomal exocytosis and this process potentiates inflammatory cytokine expression. Impairment of this process in Nox2^-/-^ and LysM-Cre Nox2-flox mice is initially beneficial, with reduced cytokine levels and improved glucose tolerance reflecting the costs associated with clearing dead adipocytes. However, over time this leads to hepatosteatosis and insulin resistance (Coats et al. 2017).

Interestingly, the altered macrophage phenotype in obesity also manifests in altered mitochondrial kinetics. One recent study (Brestoff et al. 2021) demonstrated that under homeostatic conditions macrophages acquire mitochondria from adipocytes in vivo. This uptake, mediated by heparin sulphate, is diminished in obesity. Astonishingly, inhibition of this process resulted in decreased energy expenditure and increased weight gain and glucose intolerance, regardless of food intake and physical activity (Brestoff et al. 2021). Taken collectively, the evidence above indicates that the dysfunction of macrophages during prolonged obesity are worthy of consideration and are likely to be a consequential factor in the human condition.

### Other innate immune cells in obesity

Though macrophages comprise most of the immune cell compartment in adipose tissue, dendritic cells (DCs) (Bertola et al. 2012), neutrophils (Elgazar-Carmon et al. 2008), mast cells (Liu et al. 2009), and NK cells (Lee et al. 2016), also accumulate during weight gain. Other subsets, like innate lymphoid cells (ILCs) may play roles in the induction of obesity from sites other than the adipose tissue (Sasaki et al. 2019). The accumulation of DCs in adipose tissue and the liver during high fat diet-feeding is associated with macrophage recruitment to both organs by gain and loss of function experiments (Stefanovic-Racic et al. 2012). DC deletion using *Flt3l*-deficient mice renders mice resistant to HFD-induced obesity, insulin resistance, and hepatic steatosis. Despite these mice exhibiting increased caloric intake, heightened activity and metabolic rate mitigated the effects of overnutrition (Stefanovic-Racic et al. 2012). Though this would suggest that DCs play a deleterious role in obesity, Macdougall and colleagues (Macdougall et al. 2018) revealed that VAT conventional DCs subsets actively suppress AT inflammation and promote insulin sensitivity in homeostatic lean conditions via PPARγ and the Wnt/β-catenin pathway. Disruption of these pathways with chronic overnutrition leads to DC activation, adipo-inflammation and insulin insensitivity (Macdougall et al. 2018).

Like DCs, neutrophils also infiltrate the liver and adipose tissue during high-fat feeding and help orchestrate subsequent immune cell infiltration (Elgazar-Carmon et al. 2008; Talukdar et al. 2012). Unlike DCs, neutrophils also are more direct in their contribution to obesity pathology. Mice lacking neutrophil elastase, a neutrophil protease, have a marked reduction in adipose tissue neutrophil and macrophage infiltration, higher glucose tolerance, and increased insulin sensitivity in response to HFD, mediated partially by degradation of insulin receptor substrate 1 (IRS1) (Talukdar et al. 2012).

Notably, reports on eosinophil abundance in murine adipose tissue during the lean-to-obese switch varies, with some groups citing reductions (Bolus et al. 2018a; Ding et al. 2016) and others reporting increases (Lee et al. 2018). However, eosinophils appear to fulfil beneficial function adipose tissue, suppressing obesity, sustaining alternatively activated macrophages through IL-4 secretion, and thereby promoting glucose sensitivity (Wu et al. 2011). However, the extent to which eosinophils are causal in these effects is questionable, given the report that IL-5-mediated restoration of eosinophils does not rescue the metabolic phenotype (Bolus et al. 2018b). Eosinophils may also promote beiging of WAT and enhance thermogenesis in a *Klf3-restricted* manner (Knights et al. 2020). Moreover, eosinophils, along with their IL-33-sensitive lymphoid counterparts ILC2s, are maintained in adipose tissue by sympathetic nerve signals and suppressed by the dearth of IL-33 that comes with obesity (Ding et al. 2016).

Diminished ILC2 responses are a characteristic of human and murine obesity (Brestoff et al. 2015). ILC2s have also been suggested to play an active role in WAT beiging; IL-33-mediated ILC2 production of methionine-enkephalin (met-enk) peptides were reported to induce beiging in vivo (Brestoff et al. 2015). However, the extent to which this beiging is functionally significant remain unclear as the effect of this beiging on energy expenditure was not assessed. Additionally, the direct action of opioid peptides such as met-enk on any type of adipose tissue (BAT or WAT) remains incompletely understood. When delivered systemically, opioids can induce respiratory suppression and malaise, which in turn is concomitant with anorexia and weight loss; the latter of which is also associated with beiging. An investigation into the role of ILCs in DIO indicates that small-intestinal ILC2s, but not WAT ILC2s, are involved in the induction of obesity (Sasaki et al. 2019). Though intriguing, it is unclear the extent to which the adoptive transfer approaches accurately reflect natural physiology, and the mechanism by which these ILC2s may mediate this effect has yet to be elucidated. It was thought that genetic deficiency of mast cells also protects against DIO and glucose intolerance (Liu et al. 2009). However, subsequent studies using more refined mouse models could not replicate these findings (Chmelař et al. 2016; Gutierrez et al. 2015). NK cell cytokine signalling drives M2-like to M1-like polarisation and the induction of HFD-induced insulin resistance (Lee et al. 2016; Wensveen et al. 2015).

## Role of stromal cells in adipo-inflammation

When considering how intra-adipose cellular interactions mediate obesity, we must not forget that adipose tissue depots constitute bona fide organs, comprising heterogeneous cell subtypes, including adipocytes, immunocytes, nerves and stromal cells. Until relatively recently, the contribution of the stromal cell compartment to mediating adipo-inflammation has been mostly overlooked. Published side by side, two reports from the laboratories of Diane Mathis (Spallanzani et al. 2019b) and David Artis (Mahlakõiv et al. 2019) probe this gap in our knowledge. Both groups sought to identify the cellular sources of IL-33; a cytokine negatively correlated with BMI (Hasan et al. 2014) and with a regulatory role in adipose tissue inflammation and the associated metabolic dysfunction (Han et al. 2015). Mathis and colleagues (Spallanzani et al. 2019b) used single-cell RNAseq and revealed substantial stromal cell heterogeneity and revealed mesenchymal stromal cells as major IL-33 producers. Further, they suggested that IL-33 sensitive VAT regulatory T cells (Tregs) are central to a negative feedback loop that suppresses IL-33 producing stromal cells. Moreover, they demonstrate that this loop is disrupted in obesity (Spallanzani et al. 2019b). However, the extent to which this loop contributes to obesity, remains unclear as the conditional loss of function of IL-33 from mesenchymal stromal cells has no impact on body weight nor adiposity.

In parallel, Artis et al. (Mahlakõiv et al. 2019) used flow cytometry and RNA-seq to attribute IL-33 production primarily to adipose stem and progenitor cells (ASPCs), a population that largely overlaps with the IL-33 producing population in the Mathis paper. Also, they identified a regulatory signalling axis in which ASPC-derived IL-33 promotes ILC2 activation, which in turn produce type 2 cytokines to maintain an anti-inflammatory milieu. The authors also point to their findings of high weight gain in high-fat diet-fed Il33-deficient mice relative to wild type to support the role of IL-33 in WAT homeostasis (Mahlakõiv et al. 2019). However, the lack of a more specific conditional knockout mouse models in their study means it is difficult to directly link ASPC IL-33 production to suppression of weight gain. Interestingly, the authors showed that ASPCs express IL-33 in humans (Mahlakõiv et al. 2019), suggesting that the mechanism may be relevant in human disease.

## Sympathetic Neuroimmune regulation of metabolism

Innate and adaptive immune cells express both alpha and beta adrenergic receptors (Scanzano & Cosentino 2015), thus are sensitive to sympathetic adrenergic signalling. However, expression of the β2 adrenergic receptor, and thus catecholamine sensing, is highest and seemingly most consequential among cells of the myeloid lineage (Heng et al. 2008). B2 adrenergic signalling in monocytes/macrophages has been shown to promote activation (Noh et al. 2017), pro-inflammatory cytokine production (Tan et al. 2007), M2-like polarisation (Grailer et al. 2014), and chemotaxis (Grisanti et al. 2016), in varying contexts. However, it appears their catecholamine catabolism may be more consequential.

In the context of obesity, research has primarily attributed macrophages roles in the consequences of obesity; the associated adipo-inflammation and their contribution to insulin resistance and type 2 diabetes. The idea that macrophages could play a more direct role in organismal energy metabolism and adiposity was controversial until the latter part of the last decade. Two 2017 papers were the first to show that norepinephrine catabolising ATMs populations regulate organismal adiposity. A study by Pirzgalska and colleagues identified SAMs (sympathetic neuron-associated macrophages) as cellular mediators of noradrenaline clearance and contributors to the obese state (Pirzgalska et al. 2017). SAMs were identified in subcutaneous WAT and BAT as a unique macrophage population, distinct from ATMs and microglia, characterised by the functional expression of the metabolic machinery necessary for the uptake (SLC6A2) and degradation (MAOA) of norepinephrine. Loss of Slc6a2 function in obese mice attenuates obesity, promotes lipolysis, and restores thermogenesis. SAMs are also present in human sympathetic ganglia, possessing the same machinery (MAOA, SLC6A2), suggesting the mechanism is evolutionarily conserved, and therefore could be a viable anti-obesity therapeutic target.

Simultaneously, Camell et al. reported detecting the same nerve-associated macrophages in VAT. Here, they suggested SAMs could be responsible for their observation that ATM inflammasome activation was responsible for the age-associated reduction in lipolysis. Mechanistically, the macrophage-secreted and NLRP3-regulated growth differentiation factor 3 (GDF3) mediated this lipolytic effect. Using inflammasome-activated macrophage-VAT co-cultures they also showed that MAOA, which is upregulated with ageing, negatively regulated noradrenaline-induced lipolysis (Camell et al. 2017).

In what started as an investigation into the role of macrophages in Rett syndrome, Wolf and colleagues ended up also demonstrating a central role for macrophages in energy expenditure. Targeted deletion of *Mecp2* (an essential mutated gene in Rett syndrome) in macrophages using the Cre-Lox system resulted in spontaneous obesity in normal diet-fed mice, which was accelerated with high-fat diet feeding. This was associated with significantly higher fat mass and lower lean mass, in comparison to control littermates. Macrophage-restricted *Mecp2* deletion impaired BAT innervation, noradrenaline production, and thermogenesis, likely dependent on PlexinA4 expression (Wolf et al. 2017). Conversely, another study reported that macrophage-specific deletion of insulin receptor substrate 2 induces increased BAT and WAT browning, increased sympathetic innervation of BAT, and resistance to obesity (Rached et al. 2019). Collectively, these findings serve as loss and gain of function studies, firmly implicating macrophages in the control of sympathetic innervation of BAT.

Further contributing to the notion that macrophages could play more direct roles in metabolism regulation were the two reports, from the same lab, that alternatively activated (IL-4 stimulated) ATMs can synthesise and release catecholamines (namely norepinephrine) which promote energy expenditure through non-shivering thermogenesis in BAT and lipolysis in WAT (Nguyen et al. 2011). Moreover, they argued that these alternatively activated NE-producing ATMs were involved in the beiging and cold-induced lipolysis of WAT (Qiu et al. 2014). However, these findings were starkly refuted by Fischer and colleagues (Fischer et al. 2017) who, rather than using constitutive germline-knockouts (as in refs (Nguyen et al. 2011; Qiu et al. 2014)), instead generated bone marrow chimeric mice in which *Th*, an enzyme necessary for the production of noradrenaline, could be deleted in haematopoietic cells in an inducible manner. These chimaeras, upon *Th* deletion, did not differ from WT mice in their energy expenditure at room temperature or during cold exposure. Moreover, conditioned media from in vitro alternatively activated macrophages had no effect on thermogenesis in primary WAT or BAT, and IL-4 treatment failed to impact body weight, body composition, or energy expenditure at thermoneutrality or in cold conditions. Using multiple genetic reporter approaches and RNA-seq, the group also definitively showed that macrophages did not express *Th*, a gene necessary for catecholamine synthesis (Fischer et al. 2017), a finding validated by another group (Rached et al. 2019).

The 2017 quartet of papers (Camell et al. 2017; Fischer et al. 2017; Pirzgalska et al. 2017; Wolf et al. 2017), despite seeking to answer very different questions, converged at the same conclusion; that nerve-associated adipose tissue macrophages regulate adipose tissue mass and energy expenditure through modulation, not initiation, of sympathetic noradrenergic signalling (Figure 2). Such startling discoveries revolutionised our understanding of adipose tissue homeostasis and introduce an alternate paradigm of immunometabolic regulation which could be manipulated for therapeutic benefit. In this regard, the therapeutic success of immunotherapies, for diseases other than obesity, brings new hope to patients who suffer from this stigmatising condition.

## Concluding remarks

Our understanding of obesity has now developed to the point where it cannot be designated a simple disease of energy imbalance. The obese state is characterised by neuropathologies and immunopathologies which perpetuate the obese condition. Leptin, macrophages, and norepinephrine take central roles in these adipo-pathologies. Leptin is more than a centrally-acting mediator of appetite; macrophages are more than just co-opted inflammatory bystanders; norepinephrine is a potent fat burner, constrained by SAMs that accumulate in obesity. Our advances in understanding of the neuroimmune mechanisms underlying obesity have opened the door to therapies targeting these mechanisms and a number of novel anti-obesity drugs. The pre-clinical evidence gathered so far strongly indicates that anti-obesity drugs and immunotherapeutics that curtail the metabolic consequences of obesity will be a feature of the therapeutic landscape in the years to come.
